# Genetic Susceptibility to Vitiligo: GWAS Approaches for Identifying Vitiligo Susceptibility Genes and Loci

**DOI:** 10.3389/fgene.2016.00003

**Published:** 2016-02-01

**Authors:** Changbing Shen, Jing Gao, Yujun Sheng, Jinfa Dou, Fusheng Zhou, Xiaodong Zheng, Randy Ko, Xianfa Tang, Caihong Zhu, Xianyong Yin, Liangdan Sun, Yong Cui, Xuejun Zhang

**Affiliations:** ^1^Institute and Department of Dermatology, The First Affiliated Hospital, Anhui Medical UniversityHefei, China; ^2^Department of Dermatology, The Second Affiliated Hospital, Anhui Medical UniversityHefei, China; ^3^Department of Biochemistry, University of New MexicoAlbuquerque, NM, USA; ^4^Department of Genetics and Renaissance Computing Institute, University of North Carolina at Chapel HillChapel Hill, NC, USA; ^5^Department of Dermatology, China-Japan Friendship HospitalBeijing, China

**Keywords:** Vitiligo, GWASs, genetic susceptibility, genes, loci

## Abstract

Vitiligo is an autoimmune disease with a strong genetic component, characterized by areas of depigmented skin resulting from loss of epidermal melanocytes. Genetic factors are known to play key roles in vitiligo through discoveries in association studies and family studies. Previously, vitiligo susceptibility genes were mainly revealed through linkage analysis and candidate gene studies. Recently, our understanding of the genetic basis of vitiligo has been rapidly advancing through genome-wide association study (GWAS). More than 40 robust susceptible loci have been identified and confirmed to be associated with vitiligo by using GWAS. Most of these associated genes participate in important pathways involved in the pathogenesis of vitiligo. Many susceptible loci with unknown functions in the pathogenesis of vitiligo have also been identified, indicating that additional molecular mechanisms may contribute to the risk of developing vitiligo. In this review, we summarize the key loci that are of genome-wide significance, which have been shown to influence vitiligo risk. These genetic loci may help build the foundation for genetic diagnosis and personalize treatment for patients with vitiligo in the future. However, substantial additional studies, including gene-targeted and functional studies, are required to confirm the causality of the genetic variants and their biological relevance in the development of vitiligo.

## Introduction

Vitiligo is a relatively common skin disease, and is an acquired pigmentary disorder characterized by areas of depigmented skin resulting from loss of epidermal melanocytes. The prevalence of this disease varies from 0.2% to 1% in various global populations without sex predilection (Spritz, 2008). The pathogenesis of vitiligo remains elusive, although many theories such as autoimmune hypothesis, genetics theory, reactive oxygen species model, zinc-α2-glycoprotein deficiency hypothesis, viral theory, intrinsic theory and biochemical, molecular and cellular alterations accounting for loss of functioning melanocytes in vitiligo were elaborated to clarify vitiligo pathogenesis and showed that it was a multifactorial disease involving many different interactions (Mohammed et al., 2015). In this review, we mainly summarize the recent studies in the genetics of vitiligo through genome-wide association studies (GWASs), with a focus on the susceptibility genes or loci (**Table 1**) that have been identified to date, which implicate important pathways in the pathogenesis of vitiligo.

**Table 1:** A summary of loci associated with vitiligo through GWAS, GWAS-MA studies up to 2015-10.

Chr	Reported Gene(s)	SNP-risk allele	Context	*P*-value	OR[95% CI]	Population	Reference
1p13.2	*PTPN22*	rs2476601-A	Intron	1.31E-07	1.39[1.23–1.57]	European	Jin et al., 2010a
1p36.23	*RERE*	rs4908760-G	Intron	7.07E-15	1.36[1.26–1.48]	European	Jin et al., 2010a
2q24.2	*IFIH1*	rs2111485-G	Intergenic	4.91E-15	1.30[NR]	European	Jin et al., 2012a
3q13.33	*CD80*	rs59374417-C	Intergenic	3.78E-10	1.34[NR]	European	Jin et al., 2012a
3q28	*LPP*	rs9851967-?	intron	8.57E-08	1.14[1.09–1.19]	Han Chinese	Tang et al., 2013
		rs1464510-T	intron	1.01E-11	1.31[1.21–1.41]	European	Jin et al., 2010a
4p16.1	*CLNK*	rs16872571-C	Intergenic	1.96E-08	1.21[NR]	European	Jin et al., 2012a
6p21.32	*C6orf10, BTNL2*	rs7758128-A	Intergenic	3.29E-16	2.19[1.80–2.65]	European	Jin et al., 2010a
		rs7758128-A	Intergenic	1.36E-09	1.5[NR]	European	Jin et al., 2011
	*BTNL2, HLA-DRA*	rs3806156-T	Intron	7.22E-19	1.42[1.32–1.54]	European	Jin et al., 2010a
6p21.33	*HLA-C, HLA-B*	rs11966200-A	Intron	1.48E-48	1.90[1.74–2.07]	East Asian	Quan et al., 2010
	*HLA*	rs9468925-?	Intergenic	2.21E-33	1.35[1.28–1.41]	East Asian	Quan et al., 2010
6p22.1	*HLA-A, HCG9*	rs3823355-T	NearGene-5	9.05E-23	1.50[1.39–1.63]	European	Jin et al., 2010a
6q15	*BACH2*	rs3757247-A	Intron	2.53E-08	1.20[NR]	European	Jin et al., 2012a
6q27	*RNASET2, FGFR1OP, CCR6*	rs2236313-T	Intron	9.72E-17	1.20[1.15–1.25]	East Asian	Quan et al., 2010
		rs6902119-C	Intergenic	9.75E-14	1.17[1.13–1.23]	East Asian	Quan et al., 2010
	*SMOC2*	rs13208776-?	Intron	8.51E-08	NR	Romanian	Birlea et al., 2010
8q24.22	*SLA*	rs853308-G	Intron	1.58E-08	1.20[NR]	European	Jin et al., 2012a
10p15.1	*IL2RA*	rs706779-A	Intron	2.78E-09	1.27[1.17–1.37]	European	Jin et al., 2010a
10q22.1	*SLC29A3, CDH23*	rs1417210-C	Intergenic	1.83E-08	1.14[1.09–1.19]	Han Chinese	Tang et al., 2013
10q22.3	*ZMIZ1*	rs11593576-?	Intron	8.31E-07	1.14[1.09–1.20]	East Asian	Quan et al., 2010
10q25.3	*CASP7*	rs3814231-G	Intron	3.56E-08	1.23[NR]	European	Jin et al., 2012a
11p13	*CD44*	rs10768122-G	UTR-3	1.78E-09	1.22[NR]	European	Jin et al., 2012a
11q14.3	*TYR*	rs1393350-G	Intron	1.60E-18	1.53[1.39–1.68]	European	Jin et al., 2010a
11q21	*TYR*	rs4409785-C	Intergenic	1.57E-13	1.34[NR]	European	Jin et al., 2012a
11q23.3	*CXCR5, DDX6*	rs638893-C	Intergenic	2.47E-09	1.22[1.14–1.30]	Han Chinese	Tang et al., 2013
12q13.2	*PMEL, IKZF4*	rs10876864-G	NearGene-5	8.07E-12	1.18[1.13–1.24]	Han Chinese	Tang et al., 2013
	*IKZF4*	rs2456973-C	Intron	2.75E-14	1.29[NR]	European	Jin et al., 2012a
12q24.12	*SH2B3*	rs4766578-T	Intron	3.54E-18	1.32[NR]	European	Jin et al., 2012a
14q12	*GZMB*	rs8192917-G	Missense	3.44E-08	1.28[1.17–1.39]	European	Jin et al., 2010a
		rs2273844-A	NearGene-5	6.78E-08	1.27[1.17–1.39]	European	Jin et al., 2010a
15q13.1	*OCA2, HERC2*	rs1129038-C	UTR-3	3.91E-08	1.22[NR]	European	Jin et al., 2012a
16q12.2	*KIAA1005*	rs3213758-A	Missense	6.20E-11	2.77[2.04–3.76]	Korean	Cheong et al., 2013
16q24.3	*MC1R*	rs9926296-A	Intron	1.82E-13	1.27[NR]	European	Jin et al., 2012a
19p13.3	*TICAM1*	rs6510827-T	Intron	8.80E-08	1.19[NR]	European	Jin et al., 2012a
21q22.3	*UBASH3A*	rs11203203-A	Intron	1.26E-09	1.27[1.18–1.38]	European	Jin et al., 2010a
22q13.1	*C1QTNF6*	rs229527-T	Missense	2.21E-16	1.38[1.28–1.50]	European	Jin et al., 2010a
22q13.2	*TOB2*	rs4822024-G	Intergenic	6.81E-10	1.28[NR]	European	Jin et al., 2012a

## The Concept of Vitiligo Genetics

The earliest evidence relating to the genetic basis of vitiligo was a description provided by Addison in the year of 1855, Addison presented a patient with idiopathic adrenal insufficiency, generalized vitiligo (GV), and pernicious anemia (PA; Addison, 1855). In the 1950s, perhaps the first time to address the possible inheritance of GV, Stüttgen and Teindel described multigenerational families with multiple cases of GV and other autoimmune diseases, concluding the possibility that GV is likely an autosomal dominant inheritance disease (Teindel, 1950; Stüttgen, 1950). Many subsequent studies around the world confirmed frequent clustering of vitiligo cases within families (Alkhateeb et al., 2003; Zhang et al., 2004b; Sun et al., 2006; Zhang et al., 2009). The genetics of vitiligo cannot be explained by simple Mendelian genetics, and it is characterized by incomplete penetrance, multiple susceptibility loci, and genetic heterogeneity factors. Interestingly, many of the risk loci that have been identified are shared between vitiligo and other autoimmune diseases by GWASs (**Table 2**), implying that common molecular pathways exist among various autoimmune disorder processes.

**Table 2:** Vitiligo risk loci involved in pathways and shared with other autoimmune diseases.

Function	Chromosome	Genes	Other autoimmune disease#
HLA regulation for vitiligo	6p21	HLA region	PS, SLE, RA, T1D, IBD, CD
Immunoregulatory genes for vitiligo	1p13.2 2q24	*PTPN22* *IFIH1*	RA, SLE T1D, GD, MS, Lupus
	4p16.1	*CLNK*	Gout
	6q15	*BACH2*	Asthma, CD, MS, T1D
	6p21.3	*BTNL2*	T1D, RA, SLE, PS, GD
	6q27	*CCR6*	IBD
	8q24	*SLA*	ATD, ALL
	10p15	*IL2RA*	T1D, RA, SLE
	11q23.3	*CXCR5*	CC, SLE, MS
	12q24	*SH2B3*	T1D, RA, Lupus
	12q13	*IKZF4*	T1D, AA
	21q22.3	*UBASH3A*	SLE
Melanocyte related genes for vitiligo	6q27 10q22.3	*FGFR1OP* *ZMIZ1*	CD, GD T2D, CD, IBD
	15q13.1	*OCA2*	AS, GDD, ASD
Apoptotic and cytotoxic genes	14q11.2 10q22.1	*GZMB* *SLC29A3*	JIA, BD HS
	10q25	*CASP7*	T1D, RA
Susceptibility loci with unknown functions for vitiligo	3q28 22q13.1 6q27	*LPP* *C1QTNF6* *SMOC2*	RA GD, T1D AITD

*PS, Psoriasis; SLE, Systemic lupus erythematosus; RA, Rheumatoid arthritis; T1D, Type I diabetes; T2D, Type II diabetes; IBD, Inflammatory bowel disease; CD, Crohn’s disease; GD, Graves’ disease; CC, Colorectal cancer; MS, Multiple sclerosis; AS, Angelman syndrome; GDD, Global developmental delay; ASD, Autism spectrum disorder; ALL, Acute lymphoblastic leukemia; JIA, Juvenile idiopathic arthritis; HS, H syndrome; BD, Behcet’s disease; AITD, Autoimmune thyroid disease; AA, Alopecia areata.*

## The Genetic Approaches for Vitiligo

Over the past several decades, a large number of genes and genomic regions involved in vitiligo susceptibility have been revealed through linkage analysis and candidate gene studies (Spritz, 2012). Candidate gene association studies are best suited to detect genetic signals that represent relatively common causal variants with modest effect sizes. Moreover, candidate gene association studies are relatively easy to carry out, usually involving a simple comparison of allele frequencies in cases and controls. At least 33 different candidate genes for vitiligo have been reported on the basis of such studies (Birlea et al., 2011). However, such studies are usually subject to false positive results because of the ethnic differences in case-control analyses, inadequate statistical power and statistical fluctuation, and inadequate correction for multiple testing both within and across studies.

Genome-wide linkage study is a method, when executed correctly, identifies genetic loci of vitiligo in multiplex families. These studies help to determine the position of the genetic marker inherited together with a specific disease. Genome-wide linkage studies in the Caucasian population multiplex vitiligo families identified additional linkage signals on chromosomes 7, 8, 9, 11, 13, 17, 19, and 22 (Fain et al., 2003; Spritz et al., 2004). In addition, this parallels with the genetic linkage studies of vitiligo in Chinese Han population which detected linkage signals on chromosomes 1, 4, 6, 14, and 22 (Chen et al., 2005; Liang et al., 2007). Normally, genetic loci discovered through genome-wide linkage studies encompass several megabases. The diversity of proposed regions has created a challenge in fine mapping.

**FIGURE 1:**
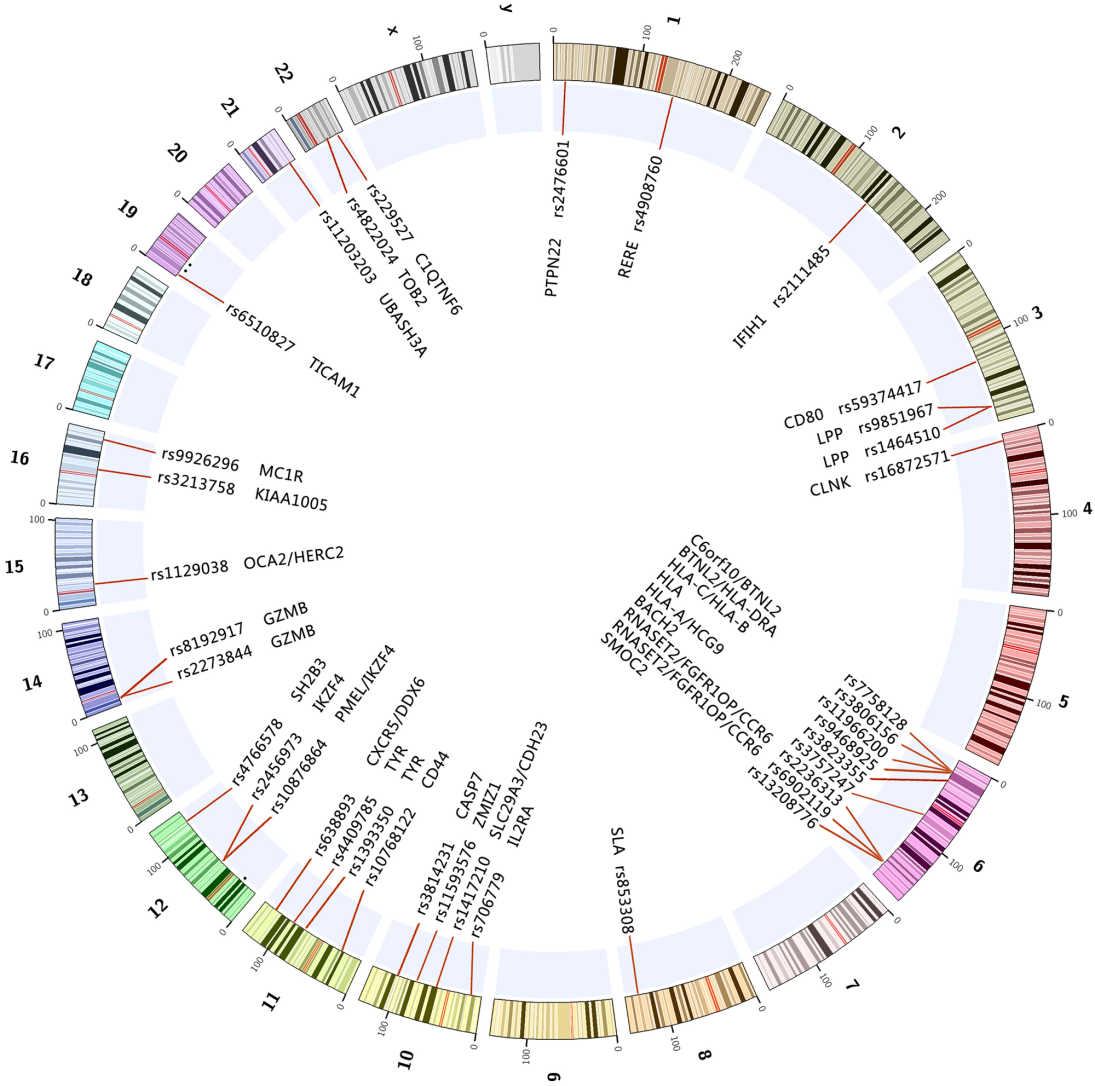
**The reported genes and loci on the chromosomes associated with vitiligo through GWAS and GWAS meta-analysis up to 2015-10.** All of the above marked regions and genes on the chromosomes achieved genome-wide significance (*p* < 5E-8) in at least one study.

Genome-wide association studies is a high through put technology, capable of “pin-pointing” disease-causing genes. Since 2005, GWAS has been proved to be the most powerful and efficient study design thus far in identifying genetic variants that are associated with complex diseases. More than 1000 types of complex diseases and traits have been investigated by this approach. Since 2010, several GWASs have been performed in patients with vitiligo in various ethnic populations (Birlea et al., 2010; Quan et al., 2010; Jin et al., 2011, 2010a, 2012a; Cheong et al., 2013), these studies have confirmed genetic associations of almost 40 genes and loci with vitiligo during the past 5 years (**Table 1**; **Figure 1**). Recently, Next-Generation DNA Re-Sequencing and exome sequencing analysis have also been used for identifying variants of genes for vitiligo (Jin et al., 2012b).

## The Susceptible Genes for Vitiligo from GWAS

### Human Leukocyte Antigen Genes

The human leukocyte antigen (HLA) is the most gene-dense region of the genome, encoding more than 120 functional genes in humans which are distributed over a 3.6 Mbp region. Many previous studies have found genes in the *HLA* region associated with vitiligo, such as *HLA-A^∗^02*, *HLA-A^∗^30*, *HLA-B^∗^13*, *HLA-C^∗^0602*, *HLA-DRB1^∗^04*, *HLA-DRB1^∗^07* and *HLA-DQB1^∗^03* (Tastan et al., 2004; Zhang et al., 2004a; Liu et al., 2007). GWASs for vitiligo have detected major association signals in the MHC on chromosome 6p21.3 in Caucasian and Chinese populations, and there are some specific associations differed between the two populations. In Caucasians, two major association peaks in MHC region were detected, the peak in class I gene region, principally between *HLA-A* and *HCG9*, and in the class II region, mainly between HLA-DRB1 and HLA-DQA1, in linkage disequilibrium (LD) with HLA-DRB1^∗^04 (Jin et al., 2010a). In the Chinese population study, the major MHC association signal was in the class III gene region, though there was also some evidence for independent association in the class II region (Quan et al., 2010). In Chinese Han population, GWAS for vitiligo identified two independent association signals (rs11966200 and rs9468925) within the *HLA* region. Further analyses have suggested that the strong association at rs11966200 might reflect the reported association of the *HLA-A^∗^3001*, *HLA-B^∗^1302*, *HLA-C^∗^0602* and *HLA-DRB1^∗^0701* alleles and the association at rs9468925 might represent a previously unknown *HLA* susceptibility allele (*HLA-C/HLA-B*) (Quan et al., 2010). Additional studies found that rs9468925 is associated with clinical features of GV (Liu et al., 2012), and more importantly, rs9468925 in *HLA-C/HLA-B* is associated with both psoriasis (PS) and vitiligo (Zhu et al., 2011), providing first important evidence that two major skin diseases share a common genetic locus in the MHC, and revealing that this genetic locus may share the same molecular mechanism for PS and vitiligo. Genome-wide analysis has identified rs532098 in the vicinity of *HLA-DRB1-DQA1*, showing suggestive evidence of the age of onset for GV (Jin et al., 2011). All evidences shown here clearly suggest that HLA genes represent attractive therapeutic targets for vitiligo pathogenesis.

### Immunoregulatory Genes for Vitiligo

Vitiligo is a common autoimmune disease, around 20% of vitiligo patients manifest concomitant occurrence of other autoimmune diseases, particularly autoimmune thyroid disease (AMD), rheumatoid arthritis (RA), late-onset type I diabetes (T1D), PS, PA, systemic lupus erythematosus (SLE), and Addison’s disease (AD; Birlea et al., 2010). Outside MHC region, some susceptibility genes encode immunoregulatory proteins which involve in biological pathways that are most likely influencing the development of vitiligo.

#### *CD44* and *CD80*

*CD44* encodes a cell surface glycoprotein with various functions, including a role in T cell development (Baaten et al., 2010), and is associated with lupus (Ramos et al., 2011). *CD80* encodes a surface protein on activated B-cells, monocytes, and dendritic cells that co-stimulates T cell priming (Peach et al., 1995). Vitiligo is a CD8 T cell-mediated autoimmune disease and promotes the longevity of memory T cell responses to melanoma. Studies have found that HA-specific CD8 T cells are adoptively transferred into mice expressing HA as a self-antigen in the pancreas, and HA-specific T cells proliferate in draining lymph nodes and upregulated *CD44* (Hernandez et al., 2001). GWAS meta analysis (GWAS-MA) of vitiligo has showed suggestive association of SNP rs4330287 and imputed SNP rs59374417 in *CD80*, which was confirmed by replication study and overall meta-analysis (Jin et al., 2012a). Flow cytometric analysis has found that the percentage of CD80+ monocytes are significantly increased in the vitiligo group compared with the controls (Basak et al., 2008), which may indicate alterations of monocyte function in the pathogenesis of vitiligo.

#### *SLA* and *BACH2*

GWAS-MA has identified vitiligo-associated loci *TG/SLA* and *BACH2* in European-derived white (CEU) population (Jin et al., 2012a). *TG* encodes thyroglobulin, *SLA* encodes Src-like adaptor protein, an inhibitor of T- and B-cell receptor signaling. It is not apparent what role thyroglobulin might play in vitiligo pathogenesis, suggesting association of vitiligo with the *TG/SLA* locus may derive from *SLA*, rather than *TG* (Jin et al., 2012a). Studies have suggested that *SLA* might likewise account for reported association with autoimmune thyroid disease (ATD; Tomer and Greenberg, 2004) and acute lymphoblastic leukemia (ALL; Mansha et al., 2010). The GWAS-MA for vitiligo showed suggestive association of SNPs (nt 90941239-91915693) spanning *BACH2*, particularly rs3757247, confirmed by the replication study and overall meta-analysis (Jin et al., 2012a). *BACH2* encodes a transcriptional repressor of B cells (Sasaki et al., 2000), which is a key regulator of CD4(+) T-cell differentiation that prevents inflammatory disease by controlling the balance between tolerance and immunity. Genetic polymorphisms analysis shows that *BACH2* is associated with asthma, Crohn’s disease (CD), multiple sclerosis (MS) and T1D (Roychoudhuri et al., 2013).

#### *IFIH1* and *TICAM1*

*IFIH1* and *TICAM1* are involved in the innate immune response system (Kato et al., 2006). GWAS-MA for vitiligo has shown genome-wide level association with SNP rs2111485 in *IFIH1* (Jin et al., 2012a). *IFIH1* encodes an interferon-induced RNA helicase involved in antiviral innate immune responses, associated with T1D (Smyth et al., 2006), Graves’ disease (GD) (Sutherland et al., 2007), MS (Martinez et al., 2008), PS (Li et al., 2010), and possibly lupus (Gateva et al., 2009). *TICAM1*, also known as TIR domain-containing adaptor-inducing IFN-b (TRIF), eventually activates transcription factors (TF), interferon regulatory factor-3 (IRF-3), NF-κB and AP-1, leading to the induction of type I interferons and inflammatory cytokines (Kumeta et al., 2014). *TICAM1* encodes a toll-like receptor adaptor molecule 1, which mediates innate immune responses to viral pathogens (Seya et al., 2009). Viral factor has been implicated in the etiopathogenesis of vitiligo, we speculate that *TICAM1* might act as a viral factor in the pathogenesis of vitiligo.

#### *PTPN22*, *UBASH3A*, and *CLNK*

*PTPN22*, *UBASH3A*, and *CLNK* are the T-cell–receptor signaling pathway genes. *PTPN22* 620W allele plays a role in autoimmune disorders, and underscores the importance of a genetically mediated autoimmune mechanism in the pathogenesis of vitiligo. Evidence shown that the *PTPN22* 1858C/T variants contribute to risk of GV in European Caucasian and Mexican populations (Laberge et al., 2008; Garcia-Melendez et al., 2014), but it does not appear to play a similar role in the Jordanian population and Turkish generalized-vitiligo patients (Alkhateeb et al., 2013; Akbas et al., 2014). A meta-analysis was conducted of the association between *PTPN22* 1858 C/T polymorphisms and vitiligo and found *PTPN22* C1858T polymorphism is associated with vitiligo susceptibility in European population (Song et al., 2013). Variants of *PTPN22* also are associated with a number of different autoimmune diseases, including RA (Begovich et al., 2004) and SLE (Kyogoku et al., 2004). There was an association between GV and nine SNPs in the region spanning *UBASH3A* on chromosome 21q22.3; of these SNPs, rs2839511 showed genome wide significance (Jin et al., 2010a). Functional prediction of the variants in non-MHC vitiligo loci identified predicted deleterious variants at *UBASH3A* confer protection from vitiligo (Jin et al., 2012a). *UBASH3A* encodes a regulator of T-cell–receptor signaling and is associated with T1D (Concannon et al., 2008), the genotype distribution of rs2277798 is significantly associated with hematuria in SLE patients (Liu et al., 2015). At 4p16.1, the GWAS-MA for vitiligo showed suggestive association of SNPs (nt 10702156–10729386) upstream of *CLNK*, including rs16872571 and several imputed SNPs, particularly rs11940117, confirmed by the replication study and overall meta-analysis. *CLNK* encodes a mast cell immunoreceptor signal transducer, a positive regulator in immunoreceptor signaling (Wu and Koretzky, 2004). Haplotype analysis has shown that the TCATTCTGA haplotype of *CLNK* is more frequent among patients with gout (Jin et al., 2015).

#### *IKZF4, IL2RA*, and *BTNL2*

*IKZF4*, *IL2RA*, and *BTNL2* are involved in T-cell activation. Two GWASs have identified *IKZF4* as one of the susceptible genes for GV (Jin et al., 2012a; Tang et al., 2013). *IKZF4*, is a critical mediator of Foxp3-dependent gene silencing in T cell reguration (Treg), interacts directly with Foxp3 and is necessary for gene silencing without affecting the expression of Foxp3 activated genes (Pan et al., 2009). *IKZF4* maybe another biological candidate gene for vitiligo and influences the development of vitiligo. Further fine mapping and function analysis required to determine the causal variants within this locus for vitiligo. Besides, studies have also found *IKZF4* is associated with T1D (Hakonarson et al., 2008) and alopecia areata (AA; Petukhova et al., 2010). There are 25 SNPs in the region of *IL2RA* (encoding the interleukin-2-receptor alpha chain) on chromosome 10p15.1, 8 of which showed genome wide significance, SNPs rs706779 and rs7090530 had the strongest association with GV (Jin et al., 2010a). Elevated serum interleukin-2-receptor levels indicate T-cell activation in GV (Honda et al., 1997; Wang et al., 2009). The variants of *IL2RA* have been shown to be associated with T1D (Vella et al., 2005), RA (Hinks et al., 2009), and SLE (Carr et al., 2009). Study identified a quantitative trait locus for vitiligo age of onset, located near *c6orf10-BTNL2* (rs7758128), a region that is also associated with GV susceptibility (Jin et al., 2011). *BTNL2* encodes an immunoglobulin superfamily membrane protein implicated in T-cell activation. Variants in *BTNL2* may play a role which involved in vitiligo susceptibility versus vitiligo age of onset. The *BTNL2* gene region has been associated with susceptibility to many other autoimmune diseases, such as T1D (He et al., 2009), RA (Cui et al., 2009), SLE (Orozco et al., 2005), PS (Feng et al., 2009), and GD (Simmonds et al., 2006).

#### *CXCR5*, *CCR6*, and *SH2B3*

*CXCR5*, *CCR6*, and *SH2B3* encode chemokine or cytokine receptors. Association analyses identified that rs638893 at 11q23.3 is associated with vitiligo in the Chinese Han population, and rs638893 is located in an intergenic region between *CXCR5* and *DDX6* (Tang et al., 2013). *CXCR5* encodes a multi-pass membrane protein that belongs to the CXC chemokine receptor family. This cytokine receptor binds to B-lymphocyte chemoattractant (BLC), and is involved in B-cell migration into B-cell follicles of spleen and Peyer patches. *CXCR5* has also been shown to have an important role in the pathogenesis of colorectal cancer (CC) (Qi et al., 2014), SLE (Zhang et al., 2014), and MS (Lill et al., 2013). At 6q27, GWAS of GV in the Chinese populations identified rs6902119 in CCR6 with genome wide significance (Quan et al., 2010). Another study showed that the most significant association SNPs rs6902119 and rs2301436 in *CCR6* were observed, SNPs rs6902119 and rs2301436 are in moderate LD, and logistic regression analysis indicated that association of GV with rs2301436 might be secondary to LD with rs6902119 (Jin et al., 2010b). *CCR6* encodes chemokine receptor 6 and is favorably expressed by immature dendritic cells and memory T cells (Schutyser et al., 2003). When binding to its ligand (CCL20), *CCR6* may result in the chemoattraction of immune cells, which might have a role in skin and mucosal surfaces under homeostatic and inflammatory conditions (Le Borgne et al., 2006). *CCR6* is also associated with inflammatory bowel disease (IBD) (Barrett et al., 2008). The GWAS-MA showed association with SNPs (nt 111708458-112906415) within and near *SH2B3*, particularly rs3184504 and imputed SNP rs4766578, located downstream, within *ATXN2* (Jin et al., 2012a). *ATXN* encodes Ataxin-2, and is causal for spinocerebellar ataxia type 2. *SH2B3* encodes a member of the *SH2B* adaptor family of proteins, which are involved in a range of signaling activities by growth factor and cytokine receptors. *SH2B3* thus seems more likely relevant to vitiligo susceptibility than *ATXN2*. *SH2B3* is also involved in the development regulation of both B and T cells, and associated with some immune diseases, including T1D (Devalliere and Charreau, 2011), RA (Coenen et al., 2009), and lupus (Li et al., 2010).

### Melanocyte Related Genes for Vitiligo

Vitiligo is one of the most common pigment disorders of the skin and hair and results from a selective destruction of melanocytes. Vitiligo patients have a progressive loss of melanocytes, predominantly in areas of skin subject to physical abrasion or at pressure points, leading to white patches appearing on the body. In normal physiological circumstances, melanin pigment is generated by the melanocytes and transferred to the surrounding keratinocytes to produce skin complexion and hair coloration (Smith and Sturm, 2010). GWASs for vitiligo have also identified some susceptible genes which showed genome-wide significant association level with the related function that influence the activity of melanocyte.

#### ZMIZ1

*ZMIZ1* locus at 10q22, encodes a protein related to protein inhibitor of activated STAT (PIAS). PIAS3, a related member of the PIAS protein family, can inhibit the transcriptional activity of microphthalmia transcription factor (MITF), which has been demonstrated to be a key regulator of melanocyte development, function and survival (Garraway et al., 2005). Another study confirmed *ZMIZ1* as a novel susceptibility locus for vitiligo and further suggested rs1408944 to be the putative causal variant that potentially interrupts TF binding and thus the transcriptional regulation of *ZMIZ1* (Sun et al., 2014). In addition, *ZMIZ1* might be associated with type II diabetes (T2D) (Matsuba et al., 2015), CD (Yang et al., 2015), and IBD (Jakobsen et al., 2014).

#### PMEL

*PMEL* encodes a melanocyte-specific type I transmembrane glycoprotein. The encoded protein is enriched in melanosomes, which are the melanin-producing organelles in melanocytes, and plays an essential role in the structural organization of premelanosomes (McGlinchey et al., 2009). Skin biopsy transcriptome analysis found that *PMEL* has a decreased expression in vitiligo lesional skin compared to vitiligo perilesional normal skin (Tang et al., 2013). In addition, the antigen-specific CD8+ T cells exhibit reactivity to modified *PMEL* peptide epitopes in HLA-A2-positive vitiligo patients (Mandelcorn-Monson et al., 2003), which also supports the notion that there is a cell-mediated immunopathologic mechanism in vitiligo.

#### TYR

GWAS identifies two SNPs (rs1847134 and rs1393350) in association with the *TYR* gene region which showed genome-wide significance with vitiligo, and haplotype analysis reveals a strong association with a block of six SNPs (rs1018528, rs10765198, rs1847134, rs1393350, rs1126809, and rs1806319) in tight LD (Jin et al., 2010a). *TYR* encodes tyrosinase, an enzyme of the melanocyte that catalyzes the rate-limiting steps of melanin biosynthesis and constitutes a major autoantigen in GV (Rezaei et al., 2007). Next-generation DNA re-sequencing identifies common variants of *TYR* and *HLA-A* that modulate the risk of GV via antigen presentation (Jin et al., 2012b). The biological interaction between *HLA-A* and *TYR* shows an apparent inverse relationship between susceptibility to GV versus malignant melanoma (Spritz, 2010), suggesting that GV may result from dysregulation of normal processes of immune surveillance against melanoma.

#### MC1R

*MC1R*, encoding the receptor protein for melanocyte-stimulating hormone (MSH), is a regulator of melanogenesis and minor vitiligo autoantigen, associating with malignant melanoma and with skin and hair color (Dessinioti et al., 2011). Expression shows *MC1R* is marked significantly different between lesional and non-lesional vitiligo skin (Kingo et al., 2007). The frequency of five *MC1R* coding region SNPs: Arg67Gln (G200A), Val92Met (G274A), Ile120Thr (T359C), Arg160Arg (C478A), and Gln163Arg (A488G) in Korean vitiligo patients and normal controls did not reach statistical significance (Na et al., 2003). However, another study shows that the Arg160Trp allele of *MC1R* gene may be able to protect against vitiligo (Szell et al., 2008). Further study need to be conducted to confirm this conclusion between vitiligo and *MC1R* coding region SNPs.

#### *RNASET2* and *FGFR1OP*

In the Chinese Han population, GWAS for vitiligo identifies a risk locus at 6q27, which contains three genes: *RNASET2*, *FGFR1OP* and *CCR6*. *RNASET2* is potentially involved in tumorigenesis and associated with human malignancies and chromosomal rearrangement. Overexpression of *RNASET2* hypersensitizes cells to oxidative stress, thus promoting cell death during peroxide exposure and stationary-phase onset (Thompson and Parker, 2009). Therefore, *RNASET2* regulates the oxidative stress and intervenes the initial pathogenic event in melanocyte destruction in vitiligo. *FGFR1OP* encodes a largely hydrophilic centrosomal protein that is required for anchoring microtubules to subcellular structures. Loss of *FGFR1OP* causes apoptosis in the G1 phase of the cell cycle, with accumulation of a 32-kDa p53 tumor suppressor isoform and NOXA and FAS transcripts, suggesting that *FGFR1OP* is necessary for cell-cycle progression and survival (Acquaviva et al., 2009). Mutations in this gene not only associate with vitiligo (Quan et al., 2010), but also are associated with CD (Yang et al., 2014), GD (Chu et al., 2011).

#### *OCA2* and *HERC2*

At 15q12–q13.1, GWAS-MA shows suggestive association of SNPs spanning *OCA2* upstream to within *HERC2*, especially SNP rs12913832 and imputed SNP rs1129038 (Jin et al., 2012a). *OCA2* encode melanocyte antigens presented by HLA-A^∗^0215, for vitiligo protection is associated with reduced functional protein, and for susceptibility to vitiligo and melanoma constitute genetic opposites, perhaps modulating immune surveillance for melanoma (Jin et al., 2012a). Mutations in *OCA2* gene associate with oculocutaneous albinism type II (Rimoldi et al., 2014) and melanoma (Hawkes et al., 2013). *HERC2* belongs to the *HERC* gene family that encodes a group of unusually large proteins, which contain multiple structural domains. Variants within *HERC2* down-regulate transcription of the *OCA2* allele in *cis* (Jin et al., 2012a). Mutations in *HERC2* are associated with the development of angelman syndrome (AS; Harlalka et al., 2013), global developmental delay (GDD), and autismspectrum disorder (AD) (Puffenberger et al., 2012).

### Apoptotic and Cytotoxic Genes for Vitiligo

Vitiligo is an acquired and progressive hypomelanotic disease that manifests as circumscribed depigmented patches on the skin. The interactions between melanocytes and other typical skin cells, particularly keratinocytes, may be an interpretation for the cause of vitiligo. Some genes have the role in gene repression, apoptosis and cell survival, inflammation, and cytotoxic cells, its possible involvement in the progression vitiligo.

#### RERE

*RERE* is the locus on chromosome 1p36.23, which encodes the arginine–glutamic acid dipeptide repeats protein. The protein is a transcriptional corepressor that is highly expressed in lymphoid cells and is thought to regulate apoptosis (Wang and Tsai, 2008). There are 40 SNPs in *RERE* were identified as having an association with GV especially rs301819 showed genome-wide significance, the most strongly associated SNPs in the *RERE* region were in tight LD; no haplotype showed a significantly stronger association than any component SNP (Jin et al., 2012a). Another SNP rs4908760 in *RERE* is the strongest associated SNP with genome-wide significance in the GWAS study (Jin et al., 2010a).

#### CASP7

At 10q25.3, GWAS-MA for vitiligo shows suggestive association of SNPs spanning *CASP7*, particularly rs3814231, which is confirmed by the replication and meta-analysis study (Jin et al., 2012a). *CASP7* encodes a member of the cysteine-aspartic acid protease (caspase) family, and sequential activation of caspases plays a central role in the execution-phase of cell apoptosis and inflammation (Lamkanfi and Kanneganti, 2010). *CASP7* also is associated with RA (Garcia-Lozano et al., 2007), and may be a candidate gene for T1D (Babu et al., 2003).

#### GZMB

Genome-wide association studies in European-derived whites have demonstrated genetic association between vitiligo and *GZMB* (Jin et al., 2012a; Jin et al., 2010a). *GZMB* is a caspase much alike serine protease that mediates two processes: immune-induced target-cell apoptosis mediated by cytotoxic T cells (CTLs), and natural killer cells and activation-induced cell death or type 2 helper T cells, which terminates the immune response (Trapani and Sutton, 2003; Devadas et al., 2006). Next-generation DNA re-sequencing has identified a direct causal role for the *GZMB* rs8192917-C-rs11539752-C haplotype (55R-94A) in the pathogenesis of GV (Ferrara et al., 2013). *GZMB* is only genetically associated with juvenile idiopathic arthritis (JIA; Donn et al., 2008) and Behcet’s disease (BD; Kucuksezer et al., 2009), which suggests the possibility that *GZMB* may be relatively specific for melanocyte-directed autoimmune susceptibility.

### Susceptibility Loci with Unknown Functions for Vitiligo

There are some vitiligo susceptible genes identified by GWAS and reach at the genome-wide significance level, but the function of these genes in the pathogenesis and development of vitiligo is still unclear.

#### TOB2

At 22q13.2, the GWAS-MA showed association with SNPs in a broad region (nt 41707054-42062822), particularly rs79008, upstream of *TOB2*, and several imputed SNPs, including rs4822024, between *ZC3H7B* and *TEF* (Jin et al., 2012a). *TOB2* locus on 22q13.2, encodes a regulator of cell cycle progression involved in T cell tolerance (Jia and Meng, 2007). However, the assignment of *TOB2* as causal remains uncertain in the pathogenesis of vitiligo.

#### SMOC2

Genome-wide association studies in an isolated European population identified *SMOC2* as a risk locus for GV (Birlea et al., 2010). However, another study shows that the variant rs13208776 in *SMOC2* gene does not play a major role in increasing the risk of vitiligo in Jordanian Arab patients (Alkhateeb et al., 2010), maybe due to the different genetic background in these two populations. In the skin, *SMOC2* is mainly present in the basal levels of the epidermis, and *SMOC2*-stimulated attachment of primary keratinocytes in culture (Maier et al., 2008). The study has also found that *SMOC2* may play a role in autoimmune thyroid disease (AITD) susceptibility as a dominant polymorphism (Alkhateeb et al., 2013).

#### KIAA1005

*KIAA1005*, also known as retinitis pigmentosa GTPase regulator-interacting protein 1-like (RPGRIP1L) gene, encodes a protein that can localize to the basal body-centrosome complex or to primary cilia and centrosomes in ciliated cells. In *KIAA1005*, the genotype and allele frequencies of 3854 G > A (1264 Asp > Asn) in vitiligo patients are significantly different compared to healthy controls. The GG frequency is lower and AA frequency is higher in vitiligo, suggesting the A allele at the *KIAA1005* G3854A may increase susceptibility to vitiligo (Cheong et al., 2013). Multiple variants of the *KIAA1005* gene have also been associated with certain clinical manifestations, particularly ciliopathies as in *DNAH5* with neurological, renal and ocular manifestations (Delous et al., 2007).

#### *SLC29A3* and *CDH23*

GWAS has revealed rs1417210 at 10q22.1 to have a strong association with vitiligo (Tang et al., 2013). This SNP is located in an LD block that contains *SLC29A3* and *CDH23*. *SLC29A3* encodes a nucleoside transporter. The encoded protein plays a role in cellular uptake of nucleosides, nucleobases, and their related correspondents. Mutations in this gene have been associated with H syndrome (Molho-Pessach et al., 2008). *CDH23* is a member of the cadherin superfamily, whose genes encode calcium dependent cell-cell adhesion glycoproteins. The encoded protein is thought to be involved in stereocilia organization and hair bundle formation. Whole-exome sequencing (WES) has identified that *CDH23* mutations cause hearing loss in Koreans families (Woo et al., 2014).

#### *LPP*, *DDX6*, and *C1QTNF6*

The *LPP* gene locus on 3q28, encodes a member of a subfamily of LIM domain proteins that are characterized by a N-terminal proline-rich region and three C-terminal LIM domains. *LPP* has also been associated with celiac disease and RA (Coenen et al., 2009). Rs638893 located in an intergenic region between *DDX6* and *CXCR5* is associated with vitiligo (Tang et al., 2013). *DDX6* encodes a member of the DEAD box protein family, which is a RNA helicase found in P-bodies and stress granules, and functions in translation suppression and mRNA degradation (Weston and Sommerville, 2006). Rs229527 in *C1QTNF6* has shown to have an association with GV (Jin et al., 2010a). *C1QTNF6* denotes the C1q and tumor necrosis factor–related protein 6 gene. *C1QTNF6-RAC2* at 22q12.3-13.1 has reached a genome-wide significant association is a novel susceptibility loci for GD (Zhao et al., 2013), and GWAS-MA also identifies *C1QTNF6* as one of risk loci for T1D (Cooper et al., 2008).

## Genes and Locus Interactions in Susceptibility to Vitiligo

In general, genes and locus only explain partial variation of heritability (Manolio et al., 2009), gene–gene (or genetic variants) interactions are strongly believed to contribute to the genetic risk of common diseases (Cordell, 2009). Transmission disequilibrium and family based association statistical tests found the SNP markers in regions 7p13, 7q11, and 9q22 were significantly associated with GV, tagging SNPs for these regions represented by rs6960920, rs734930, and rs4744411, respectively (Spritz et al., 2004). The investigators examined the potential genetic interactions for these independently identified loci using two-way tests (and three-way tests in the context of the previously identified *NLRP1* gene tagged by the rs6502867). Notably, all three SNPs showed significant interaction with the *NLRP1* gene in predicting the GV phenotype (Jin et al., 2010c). The pairwise interaction analysis between 6q27, 10q22 and the two MHC SNPs (rs11966200 and rs9468925) were performed in the Chinese Han population, but no significant genetic interaction (*P* > 0.05 after correction for multiple testing) was identified (Quan et al., 2010).

It is observed that the autoimmunity feature of vitiligo is supported through the significant linkage to the MHC region on 6p21-p22 and evidence provided for the association of *HLA-DR* with vitiligo. The epistatic interaction between rs2269577 (*XBP1*) and *HLADRB1^∗^07* was tested by using logistic regression analysis and found that the full model with both the main and interactive effects was better than the model with only the main effect to fit the data (Ren et al., 2009). Stratified association analysis of rs2269577 by *HLA-DRB1^∗^07* allele has shown that the association at rs2269577 is significant in both the patients carrying and not carrying the -DRB1^∗^07 allele. However, the association seems to be stronger in patients carrying the *HLA-DRB1^∗^07* allele (Ren et al., 2009). *XBP1* as one of biological candidate genes for vitiligo may be due to its plausible role in the development of the disease through its interaction with *HLA-DR*.

GWAS identifies *RNASET2* as a susceptible gene to vitiligo in the Chinese Han population (Quan et al., 2010), but the function of *RNASET2* in vitiligo pathogenesis or in melanocyte apoptosis is unknown. *In vitro* analyses have indicated that overexpression of *RNASET2* is inducible in cultured primary human melanocytes and keratinocytes through stressful conditions, exposure to ultraviolet irradiation, hydrogen peroxide, and inflammatory factors, respectively, and lead to increased cell apoptosis via the tumor necrosis factor receptor-associated factor 2 (TRAF2)-caspases pathway through the physical interaction of *RNASET2* with *TRAF2* (Wang et al., 2014). Hence, *RNASET2* may contribute to vitiligo pathogenesis by inhibiting *TRAF2* expression.

## Conclusion and Prospection

In the past five years, GWASs have contributed tremendously to the identification of key loci that were associated with the risk of developing vitiligo. These genes may provide novel therapeutic and prophylactic targets for new interventional approaches to treat and prevent vitiligo. Developments in this area will be exciting and influence the therapeutic approaches for the suppression of vitiligo in the future. We summarized and evaluated the importance of these loci in their respective molecular signaling pathways, and suggested new etiologic clues to vitiligo development. Considering that most of these genetic associations are restricted to moderate effects, large sample size studies are required in future investigations in order for these subtle variations to be detected. With the increasing number of GWASs being conducted, it is desirable to combine these findings across these studies to improve the statistical power. Meta-analysis of multiple GWASs improves the power to detect more associations, and to investigate the heterogeneity or consistency of these associations across different datasets and study populations. Beyond gene-association approaches, functional and gene-targeted assays, whole exome sequencing are required to identify the causal variants and understand their biological function.

## Author Contributions

XZ is the principal investigator for this review article and has contributed to the concept and planning of the article, collection of data, and reporting of the work described. CS, JG, YS, FZ, XZ, RK, XT, CZ, XY, and JD contributed to the planning of the article, collection of data, and reporting of the work described. XY, LS, and YC are the other principal investigators for this review article and have contributed to the concept of the manuscript, planning of the article, collection of data, and reporting of the work described. All authors contributed to drafting the article or revising it critically for important intellectual content.

## Conflict of Interest Statement

The authors declare that the research was conducted in the absence of any commercial or financial relationships that could be construed as a potential conflict of interest.
